# Supplementary material for: A unified connectomic target for deep brain stimulation in obsessive-compulsive disorder
Source: Nat Commun. 2020 Jul 3;11:3364. doi: 10.1038/s41467-020-16734-3 (PMC7335093; doi:10.1038/s41467-020-16734-3)
Supplement: Supplementary file 1 — Supplementary Information [file 41467_2020_16734_MOESM1_ESM.pdf]

Supplementary Information

Supplementary Information to Li et al., “A unified connectomic target for deep brain stimulation in obsessive-compulsive disorder” Nature Communications 2020.

Supplementary Figure 1

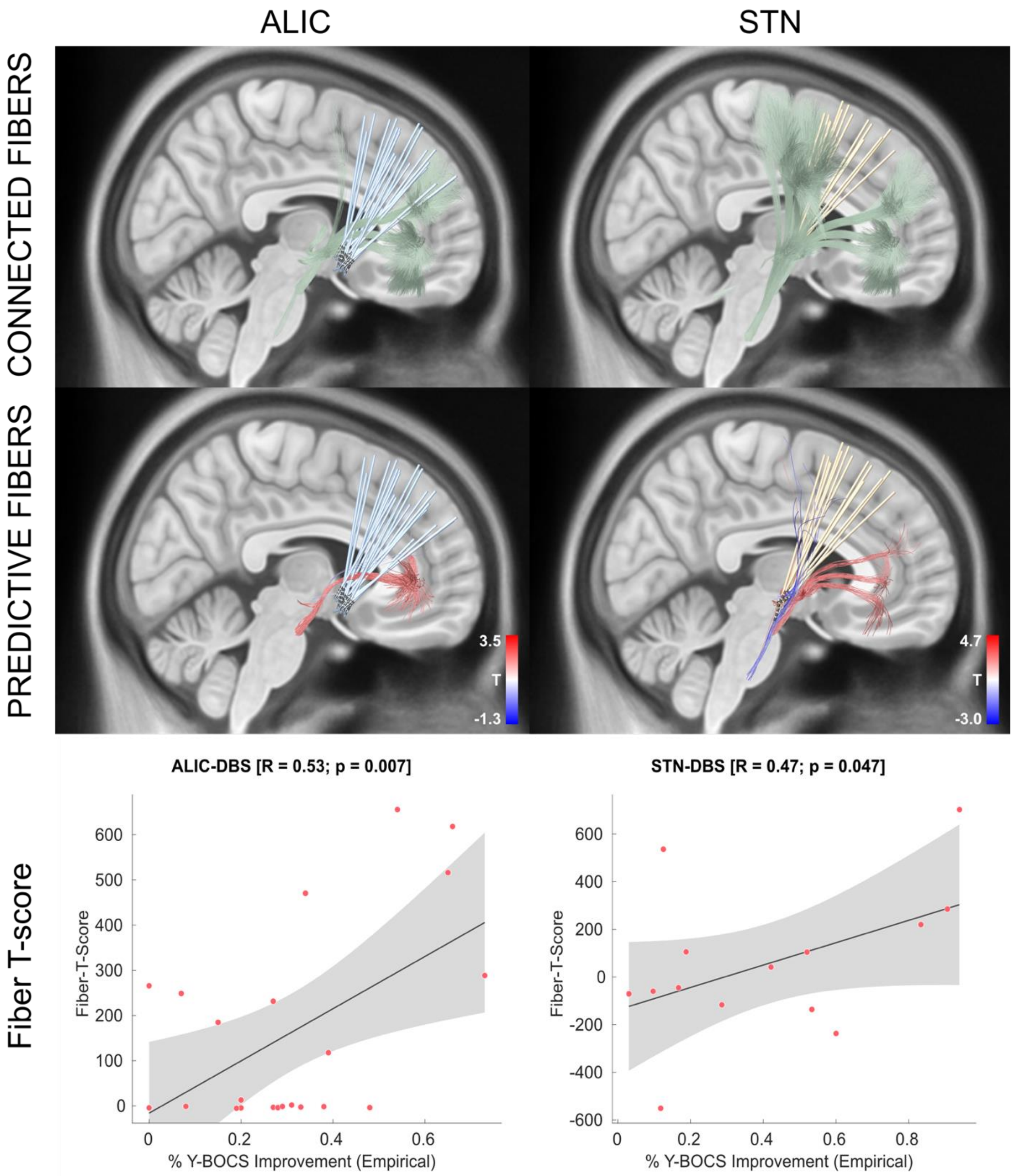

Figure 1. Replication of Figure 2 using the basal ganglia pathway atlas [1] instead of a normative connectome. This atlas is not based on diffusion-weighted imaging but on established anatomical expert knowledge and thus free of false-positive connections. While the STN target isolates three distinct bundles that traverse within the ALIC, the only predictive bundle isolated by the ALIC electrodes is the hyperdirect pathway connecting dACC and STN. In this context, it is crucial to note that connections from other regions of the prefrontal cortex (such as the ventrolateral or medial prefrontal cortex) to the STN were not represented in the atlas (and could thus represent false-negative findings, as shown by the white-matter gaps between atlas components). Gray shaded areas in the correlation plots represent 95% confidence intervals.

Supplementary Figure 2

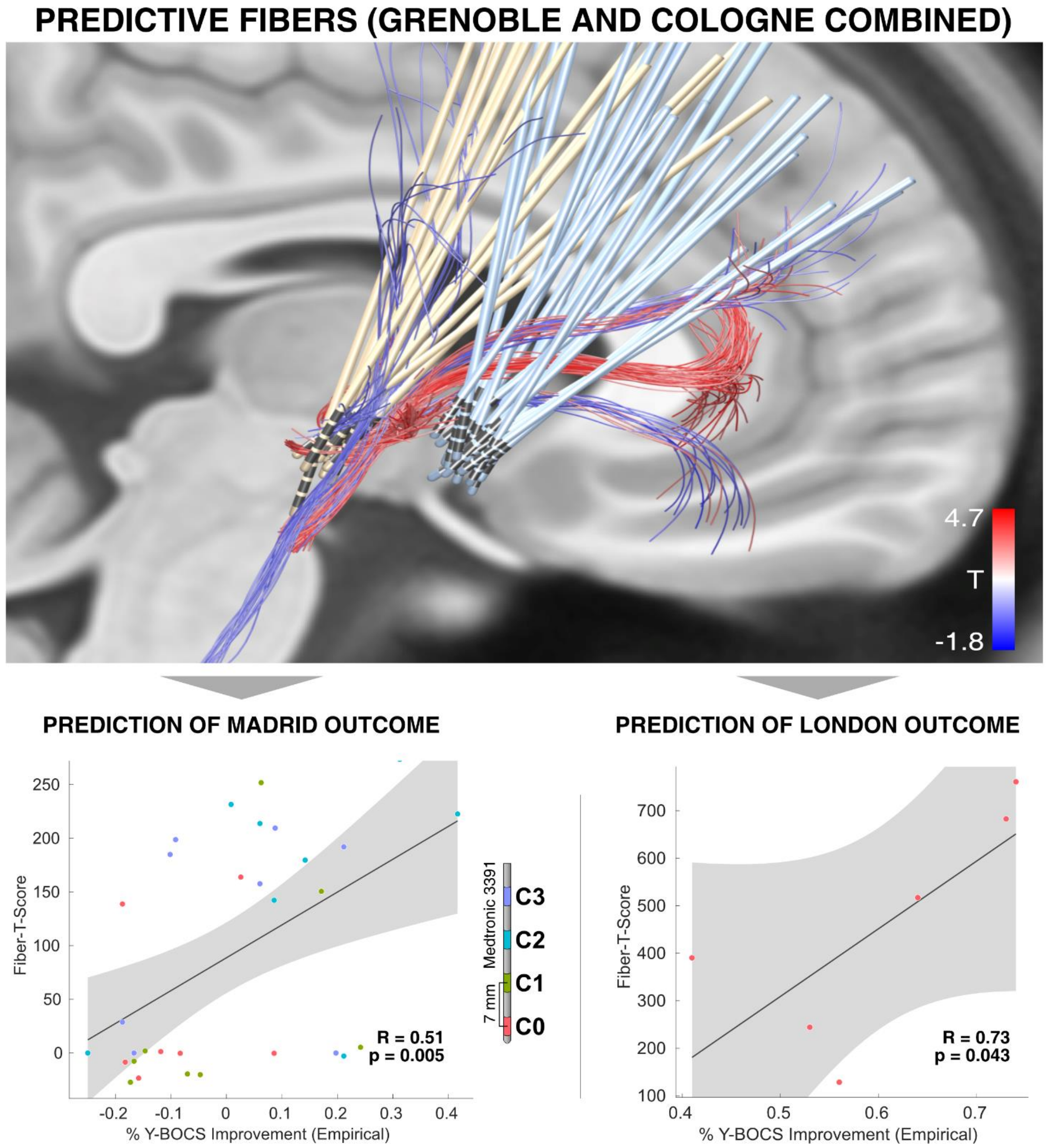

Figure 2. Replication of Figure 4 using the basal ganglia pathway atlas [1] instead of a normative connectome. This atlas is not based on diffusion-weighted imaging but on established anatomical expert knowledge and is thus free from false-positive connections. The most predictive bundle present in the atlas was the hyperdirect pathway connecting dACC and STN. In this context, it is crucial to note that connections from other regions of the prefrontal cortex (such as the ventrolateral or medial prefrontal cortex) to the STN were not represented in the atlas (and could thus represent false-negative findings, as shown by the white-matter gaps between atlas components). Gray shaded areas in the correlation plots represent 95% confidence intervals.

Supplementary Figure 3

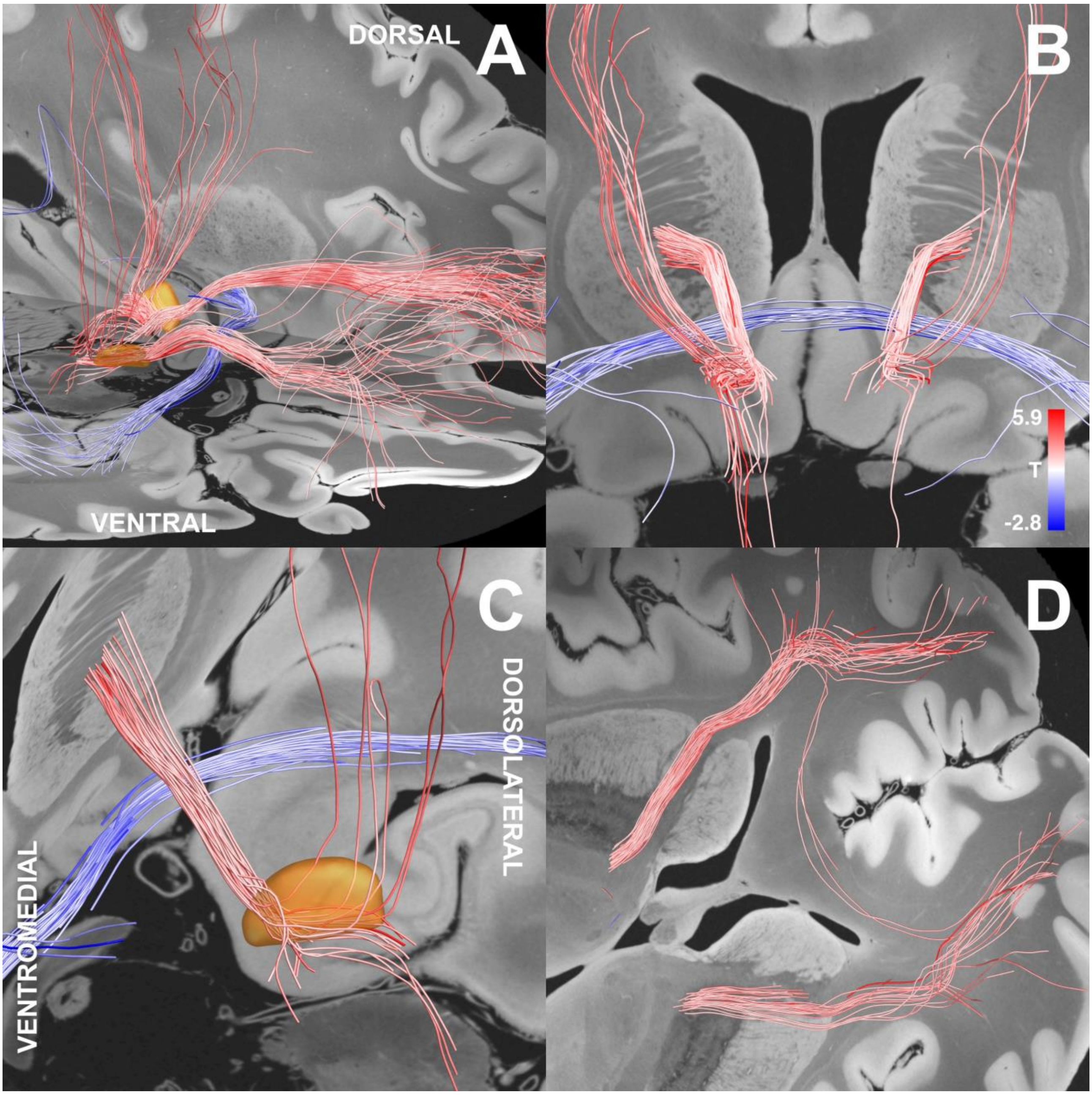

Figure 3. Anatomical definition of the final tract-target. Positively predictive fiber tracts that were discriminative in both ALIC- and STN-cohorts shown from multiple angles to further characterize them, anatomically. A) Sagittal overview with STN in orange. B) Trajectory within the internal capsule while it passes putamen and caudate. C) Close-up showing the tracts course within and below the anterior STN. D) Oblique view of the axial aspect after entering the anterior limb of the internal capsule coming in from ventrally. A 7T 100 um postmortem template is shown in the background [2].

Supplementary Methods

Literature based DBS target analysis (cf. Table 2, Figure 5)

For each publication (Table 2), pre- and postoperative average Y-BOCS scores were extracted to calculate the average percent change in Y-BOCS (difference between avg. preop- and postop-scores divided by avg. preop scores). As in all "meta-analysis" situations, due to differences in study design and reported data, we had to decide which exact values to use in some cases (below).

In 4 studies (Mallet et al. 2008 [3], Tsai et al. 2010 [4], Nuttin et al. 2003 [5], Maarouf et al. 2016 [6]), improvements were unambiguously reported. Tsai et al. [4] reported one case of hypomania induced by ventral capsule stimulation (in the vicinity of NAcc). Thus, we used the reported postoperative Y-BOCS score collected about one month after surgery to ensure consistency of pre- and postoperative medication status (sodium valproate dose was later changed to ameliorate hypomanic side effects induced by higher stimulation amplitudes). Nuttin et al. [5] reported average postoperative Y-BOCS scores that had been maintained for 21 months. In one study (Maarouf et al. 2016 [6]) that included four cases operated with different targets, we used average postoperative Y-BOCS scores of the latest follow-up in order to calculate the average percentage change in Y-BOCS. These applied to the MD/VA group of active contacts. Still, MD coordinates were used in the correlation analysis since all patients had most of their contacts in the MD region (and MD and VA nuclei are close to each other). Besides, follow-up Y-BOCS scores were not labelled as belonging to a specific target (MD vs. VA) based on the information reported. For two studies (Coenen et al. 2017 [7] and Lee et al. 2019 [8]), percentage change in Y-BOCS scores were directly reported. Coenen et al. [7] reported two OCD patients implanted to the sl-MFB target. While one patient had an improvement of 50%, the other was reported to have *over* 35% improvement. Thus, we averaged 50 and 35 % to obtain a publication based average percent change in Y-BOCS. Lee et al. [8] reported two average Y-BOCS improvements (52% for 1-year follow-up and 54% for latest follow up). We used the 1-year follow up improvement in Y-BOCS to be consistent with the other studies.

This led to average literature-based improvements in six publications for correlation analysis, while three studies (Nair et al. 2014 [9], Sturm et al. 2003 [10] and Nuttin et al. 2013 [11]) did not report Y-BOCS improvement scores. These six average improvement values were correlated with weighted overlaps between the reported average stimulation sites and the tract-target identified here. To do so, stereotactic coordinates were converted to MNI space using a novel probabilistic method [12] . A sphere of radius 3 mm was introduced at this site and heavily smoothed with a sigma of 6 mm (to allow for a weighted/distance measure with

the tract). Weighted overlap values between these smoothed volumes and the T-values of the tract-target were multiplied to derive a literature-based Fiber-T-score which was correlated with average improvement scores.

Supplementary References

1. Petersen, M. V. *et al.* Holographic Reconstruction of Axonal Pathways in the Human Brain. *Neuron* **104**, 1056-1064.e3 (2019).
2. Edlow, B. L. *et al.* 7 Tesla MRI of the ex vivo human brain at 100 micron resolution. *Sci. Data* **6**, 1–10 (2019).
3. Mallet, L. *et al.* Subthalamic Nucleus Stimulation in Severe Obsessive–Compulsive Disorder. *N. Engl. J. Med.* **359**, 2121–2134 (2008).
4. Tsai, H.-C., Chen, S.-Y., Tsai, S.-T., Hung, H.-Y. & Chang, C.-H. Hypomania Following Bilateral Ventral Capsule Stimulation in a Patient with Refractory Obsessive-Compulsive Disorder. *Biol. Psychiatry* **68**, e7–e8 (2010).
5. Nuttin, B. J. *et al.* Long-term Electrical Capsular Stimulation in Patients with Obsessive-Compulsive Disorder. *Neurosurgery* **52**, 1263–1274 (2003).
6. Maarouf, M. *et al.* Deep Brain Stimulation of Medial Dorsal and Ventral Anterior Nucleus of the Thalamus in OCD: A Retrospective Case Series. *PLOS ONE* **11**, e0160750 (2016).
7. Coenen, V. A. *et al.* The medial forebrain bundle as a target for deep brain stimulation for obsessive-compulsive disorder. *CNS Spectr.* **22**, 282–289 (2017).
8. Lee, D. J. *et al.* Inferior thalamic peduncle deep brain stimulation for treatment-refractory obsessive-compulsive disorder: A phase 1 pilot trial. *Brain Stimulat.* **12**, 344–352 (2019).
9. Nair, G., Evans, A., Bear, R. E., Velakoulis, D. & Bittar, R. G. The anteromedial GPi as a new target for deep brain stimulation in obsessive compulsive disorder. *J. Clin. Neurosci.* **21**, 815–821 (2014).
10. Sturm, V. *et al.* The nucleus accumbens: a target for deep brain stimulation in obsessive–compulsive- and anxiety-disorders. *J. Chem. Neuroanat.* **26**, 293–299 (2003).

11. Nuttin, B. *et al.* Targeting Bed Nucleus of the Stria Terminalis for Severe Obsessive-Compulsive Disorder: More Unexpected Lead Placement in Obsessive-Compulsive Disorder than in Surgery for Movement Disorders. *World Neurosurg.* **80**, S30.e11-S30.e16 (2013).

12. Horn, A. *et al.* Probabilistic conversion of neurosurgical DBS electrode coordinates into MNI space. *NeuroImage* **150**, 395–404 (2017).
