# Supplementary material for: A unified connectomic target for deep brain stimulation in obsessive-compulsive disorder
Source: Nat Commun. 2020 Jul 3;11:3364. doi: 10.1038/s41467-020-16734-3 (PMC7335093; doi:10.1038/s41467-020-16734-3)
Supplement: Supplementary file 3 — Reporting Summary [file 41467_2020_16734_MOESM3_ESM.pdf]

nature research

Corresponding author(s): Ningfei Li

Last updated by author(s): May 6, 2020

Reporting Summary

Nature Research wishes to improve the reproducibility of the work that we publish. This form provides structure for consistency and transparency in reporting. For further information on Nature Research policies, see Authors & Referees and the Editorial Policy Checklist .

Statistics

For all statistical analyses, confirm that the following items are present in the figure legend, table legend, main text, or Methods section.

n/a	Confirmed	
☐	☒	The exact sample size (*n*) for each experimental group/condition, given as a discrete number and unit of measurement
☐	☒	A statement on whether measurements were taken from distinct samples or whether the same sample was measured repeatedly
☐	☒	The statistical test(s) used AND whether they are one- or two-sided<br>*Only common tests should be described solely by name; describe more complex techniques in the Methods section.*
☒	☐	A description of all covariates tested
☒	☐	A description of any assumptions or corrections, such as tests of normality and adjustment for multiple comparisons
☒	☐	A full description of the statistical parameters including central tendency (e.g. means) or other basic estimates (e.g. regression coefficient) AND variation (e.g. standard deviation) or associated estimates of uncertainty (e.g. confidence intervals)
☐	☒	For null hypothesis testing, the test statistic (e.g. F, t, r) with confidence intervals, effect sizes, degrees of freedom and P value noted<br>*Give P values as exact values whenever suitable.*
☒	☐	For Bayesian analysis, information on the choice of priors and Markov chain Monte Carlo settings
☒	☐	For hierarchical and complex designs, identification of the appropriate level for tests and full reporting of outcomes
☐	☒	Estimates of effect sizes (e.g. Cohen's *d*, Pearson's *r*), indicating how they were calculated

Our web collection on statistics for biologists contains articles on many of the points above.

Software and code

Policy information about availability of computer code

Data collection	Microsoft Excel
Data analysis	MATLAB, Lead-DBS, Medtronic SureTune, SPM12, Advanced Normalization Tools, Lead Connectome, BRAINSFit, PaCER, SimBio, FieldTrip

For manuscripts utilizing custom algorithms or software that are central to the research but not yet described in published literature, software must be made available to editors/reviewers. We strongly encourage code deposition in a community repository (e.g. GitHub). See the Nature Research guidelines for submitting code & software for further information.

Data

Policy information about availability of data

All manuscripts must include a data availability statement. This statement should provide the following information, where applicable:

- Accession codes, unique identifiers, or web links for publicly available datasets
- A list of figures that have associated raw data
- A description of any restrictions on data availability

The DBS MRI datasets generated during and analyzed during the current study are not publicly available due to data privacy regulations of patient data but are available from the corresponding author on reasonable request.

Field-specific reporting

Please select the one below that is the best fit for your research. If you are not sure, read the appropriate sections before making your selection.

☒ Life sciences ☐ Behavioural & social sciences ☐ Ecological, evolutionary & environmental sciences

Life sciences study design

All studies must disclose on these points even when the disclosure is negative.

Sample size	N = 50 DBS patients, sample size was determined heuristically based on effect sizes in Baldermann 2019 BPS (N = 22 patients). OCD-DBS patients are a very rare clinical population, even on a global scale. Most studies performed were based on local samples of DBS centers (i.e. based on availability), most of them ranging around an N = 10-20 patients. Here, we drew inferences on sample sizes based on effect sizes of previous studies, especially Baldermann et al. 2019 BPS. Based on these, a power analysis estimated an N of ~25-30 to be necessary to perform a split-half prediction analysis. We were able to retrospectively collect a larger sample of N = 50 patients.
Data exclusions	No data was excluded
Replication	Training and cross-validation of the model was performed on two large sub-cohorts (N = 22, N = 14) and tested / replicated on two smaller cohorts (N = 6, N = 8 patients). All attempts of replication were successful.
Randomization	Participants were associated to training / test groups based on i) the center they were operated in and ii) the surgical target to which the electrodes had been implanted. Validations were performed on datasets that we obtained after the training period, by further collaborators (London, Madrid).
Blinding	This was not a cohort study comparing groups, so Blinding does not apply. Rather, we estimated relationships between electrode placements and clinical improvements following OCD-DBS surgery at various surgical targets and DBS centers.

Reporting for specific materials, systems and methods

We require information from authors about some types of materials, experimental systems and methods used in many studies. Here, indicate whether each material, system or method listed is relevant to your study. If you are not sure if a list item applies to your research, read the appropriate section before selecting a response.

Materials & experimental systems

n/a	Involved in the study	
☒	☐	Antibodies
☒	☐	Eukaryotic cell lines
☒	☐	Palaeontology
☒	☐	Animals and other organisms
☐	☒	Human research participants
☐	☒	Clinical data

Methods

n/a	Involved in the study	
☒	☐	ChIP-seq
☒	☐	Flow cytometry
☐	☒	MRI-based neuroimaging

Human research participants

Policy information about studies involving human research participants

Population characteristics	Four subcohorts from four DBS centers, all of them patients suffering from obsessive compulsive disorder and all of them retrospectively recruited. 26 of the 50 patients were female, 24 male. Cohort 1 (Cologne / ALIC DBS): 22 patients, 12 females, aged 41.7 ± 20.5 yrs. Cohort 2 (Grenoble / STN-DBS): 14 patients, 9 female, age 41 ± 9 yrs. Cohort 3 (Madrid / NAcc DBS): 8 patients (4 female), age 35.3 ± 10.4 yrs. Cohort 4 (London, combined ALIC & STN DBS): 6 patients, 1 female, age 45.5 ± 10.5 yrs.
Recruitment	Patients were retrospectively enrolled based on priorly published data.
Ethics oversight	The study was approved by the local ethics committee of Charité – University Medicine Berlin, master vote = EA2/186/18

Note that full information on the approval of the study protocol must also be provided in the manuscript.

Clinical data

Policy information about clinical studies

All manuscripts should comply with the ICMJEguidelines for publication of clinical research and a completedCONSORT checklist must be included with all submissions.

Clinical trial registration	N/A; Purely retrospective analysis of published clinical data.
Study protocol	N/A; Purely retrospective analysis of published clinical data.
Data collection	N/A; Purely retrospective analysis of published clinical data.

Outcomes	N/A; Purely retrospective analysis of published clinical data.

Magnetic resonance imaging

Experimental design

Design type	Structural Imaging was used only (no fMRI). Structural Imaging was acquired during clinical routine, not specifically for the present (retrospective) study.
Design specifications	Structural Imaging was used only (no fMRI). Structural Imaging was acquired during clinical routine, not specifically for the present (retrospective) study.
Behavioral performance measures	Structural Imaging was used only (no fMRI). Structural Imaging was acquired during clinical routine, not specifically for the present (retrospective) study. Thus, no Behavioral performance measures were taken.

Acquisition

Imaging type(s)	structural, diffusion
Field strength	3T, 1.5T
Sequence & imaging parameters	Structural Imaging: Clinical Scans in Patients performed outside of the study (retrospective data analysis)<br>Diffusion Imaging: Based on openly available database (Human Connectome Project), thus not acquired specifically for the present study.
Area of acquisition	whole brain

Diffusion MRI ☒ Used ☐ Not used

Parameters	The gradient table includes approximately 90 diffusion weighting directions plus 6 b=0 acquisitions interspersed throughout each run. Diffusion weighting consisted of 3 shells of b=1000, 2000, and 3000 s/mm2 interspersed with an approximately equal number of acquisitions on each shell within each run.

Preprocessing

Preprocessing software	LeadDBS v2
Normalization	Multispectral Normalization using Advanced Normalization Tools SyN based approach, validated in Ewert et al. 2019 NeuroImage
Normalization template	ICBM 2009b NLIN Asym (Fonov et al. 2009). This is the most accurate (0.5 mm) and most modern "MNI" template available.
Noise and artifact removal	No fMRI data was acquired. Diffusion data was preprocessed by the Human Connectome Project team at Washington University St. Louis.
Volume censoring	No censoring took place.

Statistical modeling & inference

Model type and settings	N/A – no fMRI analysis performed.
Effect(s) tested	N/A – no fMRI analysis performed.

Specify type of analysis: ☒ Whole brain ☐ ROI-based ☐ Both

Statistic type for inference (See Eklund et al. 2016)	N/A – no fMRI analysis performed. We used MRI to localize DBS electrodes only.
Correction	N/A (see above).

Models & analysis

n/a	Involved in the study
☒	☐ Functional and/or effective connectivity
☒	☐ Graph analysis
☒	☐ Multivariate modeling or predictive analysis
